# Hematopoietic Stem/Progenitor Cell Dependent Participation of Innate Lymphoid Cells in Low-Intensity Sterile Inflammation

**DOI:** 10.3389/fimmu.2018.02007

**Published:** 2018-09-05

**Authors:** Sarantis Korniotis, Thomas B. Thornley, Periklis Kyriazis, Evangelos Theodorou, Lingzhi Ma, Lisa S. Li, Efi Kokkotou, Terry B. Strom, Maria Koulmanda

**Affiliations:** ^1^Department of Medicine, Harvard Medical School and the Transplant Institute at Beth Israel Deaconess Medical Center, Boston, MA, United States; ^2^Division of Gastroenterology, Harvard Medical School, Beth Israel Deaconess Medical Center, Boston, MA, United States; ^3^Department of Surgery, Harvard Medical School and the Transplant Institute at Beth Israel Deaconess Medical Center, Boston, MA, United States

**Keywords:** sterile inflammation, CCR2, hematopoietic stem cells, innate lymphoid cells (ILC), zymosan

## Abstract

Hematopoietic stem/progenitor cells (HSPC) are characterized by their unique capacities of self-renewal and multi-differentiation potential. This second property makes them able to adapt their differentiation profile depending on the local environment they reach. Taking advantage of an animal model of peritonitis, induced by injection of the TLR-2 ligand, zymosan, we sought to study the relationship between bone marrow-derived hematopoietic stem/progenitor cells (BM-HSPCs) and innate lymphoid cells (ILCs) regarding their emergence and differentiation at the site of inflammation. Our results demonstrate that the strength of the inflammatory signals affects the capacity of BM-derived HSPCs to migrate and give rise *in situ* to ILCs. Both low- and high-dose of zymosan injections trigger the appearance of mature ILCs in the peritoneal cavity where the inflammation occurs. Herein, we show that only in low-dose injected mice, the recovered ILCs are dependent on an in situ differentiation of BM-derived HSPCs and/or ILC2 precursors (ILC2P) wherein high-dose, the stronger inflammatory environment seems to be able to induce the emergence of ILCs independently of BM-derived HSPCs. We suggest that a relationship between HSPCs and ILCs seems to be affected by the strength of the inflammatory stimuli opening new perspectives in the manipulation of these early hematopoietic cells.

## Introduction

Innate lymphoid cells (ILCs) arise from fetal progenitors or from common lymphoid progenitors (CLP) within the bone marrow. During the late stages of the fetal development, fetal hematopoietic lymphoid progenitors from the bone marrow migrate and populate different tissues giving rise to lymphoid structures (1–3). ILCs have been subdivided into three groups on the basis of development, function, patterns of cytokine production and expression of transcription factors (4, 5). Regarding group 1, the ILCs display a Th1-like cytokine signature, producing large amounts of IFN-γ and TNF-α and, are positive for the expression of T-bet (6, 7). In contrast, group 2 ILCs secrete Th2-related cytokines such as IL-4, IL-5, and IL-13 together with up-regulated expression of the transcription factor Gata-3 (4, 8–10). Group 3 ILCs produce pro-inflammatory cytokines including IL-17, IL-21, IL-22, and GM-CSF and, express the transcription factor RORgt (11–13). ILC1 and ILC3, but not ILC2, express retinoic acid and CCR7 homing receptors that are also expressed by activated T cells. Upon activation, these receptors guide the migration of activated T cells to non-lymphoid tissues (14). In contrast, ILC2s use homing receptors, in common with myeloid and certain innate cells (14).

Using a model of sterile inflammatory peritonitis, induced by intraperitoneal injection of zymosan, a toll-like receptor 2 (TLR2) ligand, we compared the consequence of induction by high- or low-dose zymosan treatment upon the nature, origin, and abundance of various immune cell types from the peritoneal cavity at 24 h (15, 16). Knowing that hematopoietic stem/progenitor cells (HSPCs) migrate to inflamed tissues (17), we investigated whether mature ILCs migrate directly to the inflammatory site or BM-HSPCs differentiate *in situ* into ILCs. To further analyze the influence of the strength of inflammation and the potential for therapeutic intervention, we analyzed the impact of treatment with alpha-1 anti-trypsin (AAT), a potent anti-inflammatory protein (18) in the low- and high-dose zymosan-induced peritonitis model at 24 h.

## Results

### Intraperitoneal injection of high- or low-dose of zymosan, a TLR2 ligand, regulates the presence and phenotype of ILCs in the peritoneal cavity

**Figure 1:**
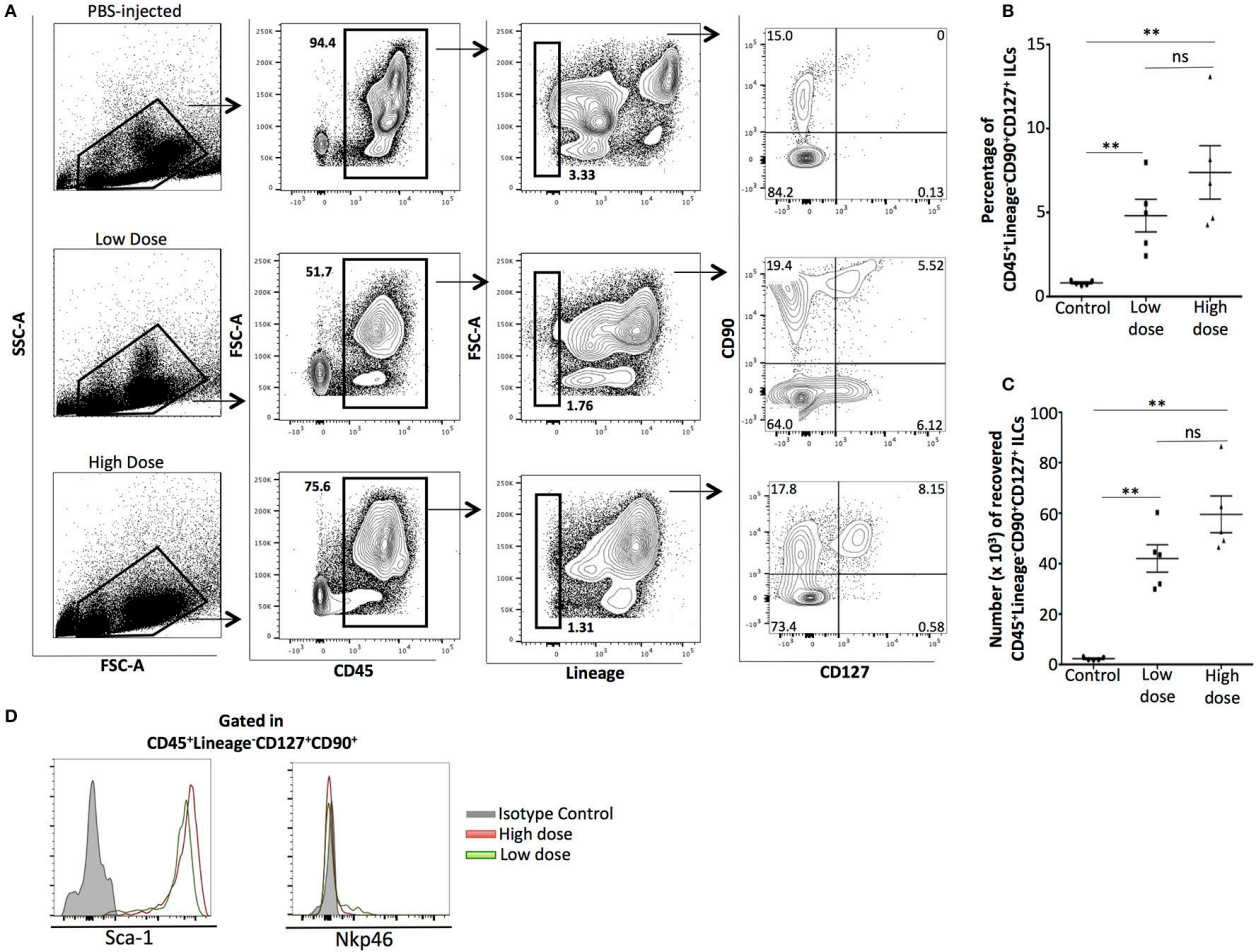
Phenotypic analysis of innate lymphoid cells recovered from the peritoneal cavity of zymosan-injected mice, 24 h post-injection. **(A)** Cells from the peritoneum of mice injected with PBS 1X (controls) or low (0.1 mg/ml) or high (10 mg/ml) dose of zymosan were recovered 24 h post-injection. Cells were stained for CD45, Lineage, CD90, and CD127. One representative flow cytometry analysis is shown from 5 different independent experiments (*n* = 5). **(B)** Significant increase in the frequency (percentage) of total ILCs (CD45^+^Lin^−^CD90^+^CD127^+^) cells emerged in the peritoneal cavity of zymosan-, but not in PBS-injected mice was observed (*n* = 5) **(C)** No significant difference observed on the total cell number of ILCs between low- and high-dose treated mice (*n* = 5), **(D)** ILCs recovered from the peritoneal cavity of both low- and high-dose of zymosan-treated mice were stained for additional markers including Sca-1 and Nkp46 and their expression level is shown (*n* = 4). **p < 0.005.

We injected C57BL/6J mice i.p with low (0.1 mg/ml) or high (10 mg/ml) doses of zymosan or PBS (Control) and the peritoneal exudate was harvested at 24 h. Both doses of zymosan induce sterile inflammation in the peritoneal cavity. The intraperitoneal cells were stained for CD45, Lineage markers (anti-CD3, anti-Ly-6G/Ly-6C, anti-CD11b, anti-B220/CD45R, and anti-Ter-119), CD127, and CD90. Consistent with the participation of ILCs in sterile inflammation (19), cells with a CD45^+^Lineage^−^CD127^+^CD90^+^ phenotype were identified in the zymosan-treated, but not in PBS-injected mice (Figure 1A). There was no significant difference in both frequency (percentage) and total cell number of ILCs between the two groups of zymosan-treated mice (Figure 1B,C). Additionally, ILCs were negative for the expression of Nkp46 (an NK cell type with some characteristics shared by group 3 ILCs) and all ILCs were Sca-1^+^ (Figure 1D).

### HSPCs are attracted to high- and low-dose TLR2-stimulated sterile inflammatory sites

**Figure 2:**
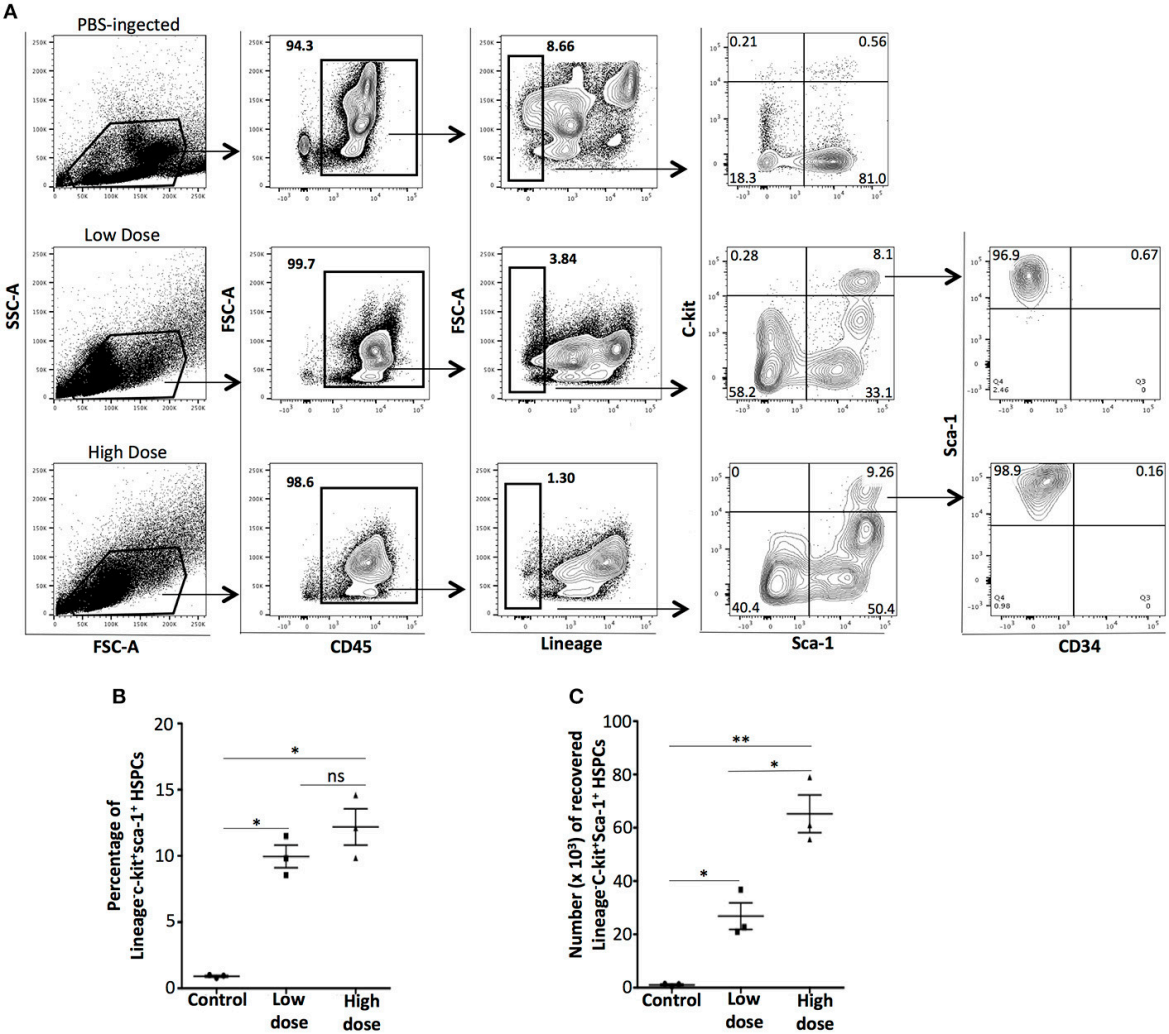
Zymosan induces the emergence of bone marrow-derived hematopoietic stem/progenitor cells to the site of inflammation. WT C57BL/6 mice were injected with high- or low-dose of zymosan and after 24 h cells in the peritoneal cavity were harvested and used for FACS analysis. **(A)** Cells from the peritoneal cavity of zymosan-injected mice were stained for CD45, Lineage, CD117 (c-kit), Sca-1, and CD34. Intraperitoneal injection of zymosan induces the emergence of a population of CD45^+^Lineage^−^c-kit^+^Sca-1^+^CD34^−^ cells, a phenotype corresponding to early hematopoietic stem/progenitor cells. One representative flow cytometry analysis is shown from 3 independent experiments (*n* = 3). **(B,C)** In both low- and high-dose of treatment, a significant increase of the percentage of the recovered HSPCs has noticed whereas the absolute total number of HSPCs in high-dose zymosan is higher compared to low-dose treated mice (*n* = 3). **p < 0.005, *p < 0.05.

It has been previously shown that bone marrow-derived HSPCs are attracted to the inflammatory environment of thioglycollate-induced peritonitis (20) and syngeneic or allogeneic organ or cell transplants (17). In the zymosan-induced model of sterile peritonitis, we examined whether the migration of HSPCs to the peritoneal cavity is dependent on the strength of the inflammatory stimulus. We probed for the presence of HSPCs in C57BL6/J mice injected i.p either with low- or high-dose of zymosan or PBS (control) 24 h post-injection. Intraperitoneal cells were stained with the anti-lineage cocktail, the hematopoietic cell lineage CD45 marker and stem cell markers such as CD117 (c-kit), Sca-1 and CD34. In both low- and high-dose of zymosan, but not in PBS-treated mice, a population of CD45^+^Lineage^−^ckit^+^Sca-1^+^CD34^−^ cells was recovered (Figure 2A). Although a similar percentage of HSPCs cells was noted in both low- and high-dose treated mice (Figure 2B), the total number of cells in mice injected with high-dose of zymosan was significantly higher (Figure 2C).

### Different requirements for HSPCs in the appearance of ILCs within sites of sterile inflammation, in response to a low- or high-dose of zymosan

**Figure 3:**
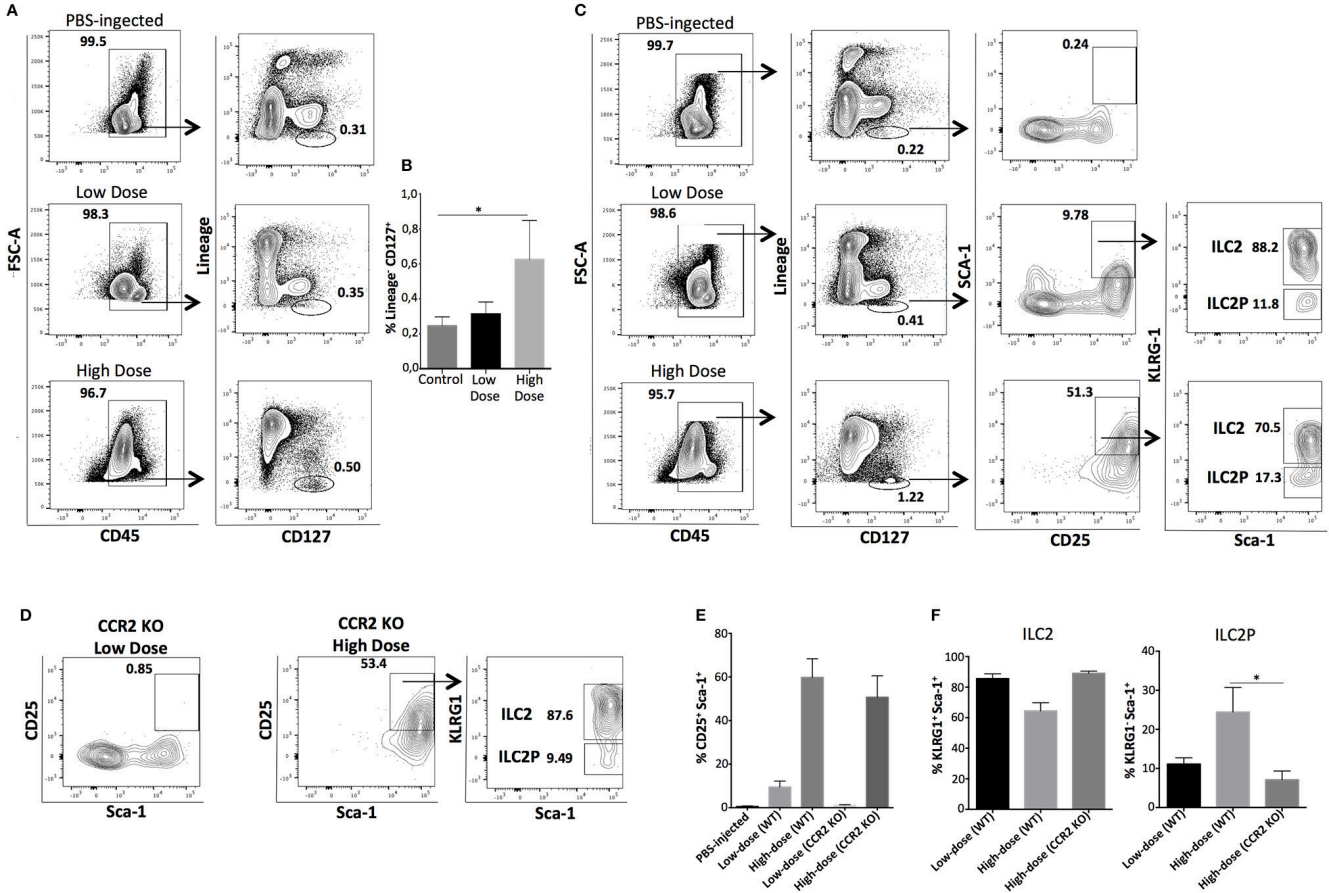
ILCs and ILC2P cells emerge to the site of inflammation independently of HSPCs in response to high-dose of zymosan treatment. WT or CCR2 KO mice were injected with 0,1 mg (low dose) or 10 mg (high dose) of zymosan and 24 h later peritoneal cells were harvested, stained for markers identifying both mature ILCs and progenitor cells of type 2 ILCs. **(A)** CCR2 KO mice where bone marrow-derived HSPCs are unable to migrate to the site of sterile inflammation, were injected either with a low- or high-dose of zymosan. Twenty-four hours post-injection, cells were recovered from the peritoneum of injected mice and stained for Lineage markers, CD45 and CD127. One representative experiment of flow cytometry analysis is shown from 4 independent experiments (*n* = 4) **(B)** Statistical analysis of the frequency of Lineage^−^CD127^+^ cells recovered from the peritoneal cavity of zymosan-injected mice (CCR2 KO recipients). The same experimental process as described in **(A)** was used to investigate the emergence of bone marrow-derived progenitor cells for type 2 innate lymphoid cells in WT mice treated either with a low- or high-dose of zymosan. **(C)** Cells were stained for CD45, Lineage markers, CD127, CD25, Sca-1, and KLRG-1 (*n* = 4). **(D)** Intraperitoneal cells were recovered from the peritoneal cavity of both high- and low-dose treated CCR2 KO mice and stained for markers of ILC2 and ILC2P as described in **(B)**. One representative experiment is shown (*n* = 4). **(E)** Statistical analysis of the frequency of CD25^+^Sca-1^+^ cells gated previously in CD45^+^Lineage^−^CD127^+^ cells of both WT and CCR2 KO mice injected either with PBS (control) or low- and high-dose of zymosan (*n* = 4). **(F)** Statistical analysis of the frequency of Sca-1^+^KLRG1^+^ and Sca-1^+^KLRG1^−^ cells from mice injected either with PBS or low- and high-dose zymosan both in WT and CCR2 KO mice (*n* = 4). *p < 0.05.

In thioglycollate-induced inflammation, HSPCs migrate to the peritoneum in a CCR2-dependent manner (20). After confirming in our study that bone marrow-derived HSPCs express CCR2 (Supplementary Figures 1A,B), we subsequently investigated whether homing of HSPCs to the peritoneal cavity in response to zymosan is also CCR2 dependent. For these experiments we used CCR2 KO mice in our low- and high-dose zymosan model. As shown in Supplementary Figure 2, zymosan at either dose was less efficient in inducing the appearance of HSPCs in the peritoneum of CCR2 KO mice compared to WT ones. Then, we asked whether attenuated migration of HSPCs in CCR2 KO mice with peritonitis had an impact on the emergence of peritoneal ILCs. CCR2 KO mice were injected i.p either with low- or high-dose of zymosan, and 24 h post-injection, cells in the peritoneal fluid were stained for targets of the Lineage cocktail, CD45, and CD127. Only the high-dose of zymosan induced the emergence of a significant population of CD45^+^Lineage^−^CD127^+^ ILCs in the peritoneal cavity in the absence of HSPCs (Figures 3A,B). The appearance of mature ILCs in the peritoneal cavity of zymosan-injected mice might result of *in situ* differentiation of innate lymphoid progenitor cells derived from the bone marrow, which might respond only to high-dose inflammatory signals. Since high-dose treatment favors mostly the emergence of ILCs of type 2, based on the expression of Gata-3 and IL-5 (Supplementary Figure 3), we sought to investigate whether high-dose treatment of zymosan induces more efficiently than low-dose, the appearance of bone marrow-derived precursors of innate lymphoid cells type 2 (ILC2P) (21). Wild-type (WT) C57BL/6J mice were injected intraperitoneally either with low- or high-dose of zymosan and cells in the peritoneal cavity were stained for Lineage markers, CD45, CD127, CD90, CD25, and KLRG1 for identification of ILC2Ps. As shown in Figure 3C, both high- and low-dose treatments induce the emergence of a Lineage^−^CD45^+^CD127^+^CD25^+^Sca1^+^KLRG1^−^ population, which corresponds to BM-derived precursor cells of ILC type 2, but not with the same intensity. Specifically, the frequency of cells expressing a CD25^+^Sca-1^+^ phenotype is significantly higher in the high-dose of zymosan treatment (Figures 3C,E,F). Additional markers such as ST2 and IL17RB have been also shown to identify specifically type 2 ILCs (10, 22–24). In our experimental setting, KLRG1^+^ ILCs recovered from the peritoneal cavity of zymosan-injected mice, were all positive for the expression of ST2 but negative for IL17RB (Supplementary Figure 4A). In order to test the dependence of these cells on HSPCs, CCR2 KO (C57BL/6J) mice were injected with high- and low-dose of zymosan and cells were stained for markers characterizing the progenitors of ILC2P and mature ILC2. The low-dose injection was unable to trigger the emergence of any of these populations where the high-dose treatment resulted in the appearance of both populations, suggesting that there might be a different dependence on HSPCs regarding the migration of ILCs to the site of inflammation (Figure 3D).

### BM-derived hematopoietic stem cells differentiate *in situ* into innate-like lymphoid cells in an inflammatory environment induced by low-dose of zymosan

**Figure 4:**
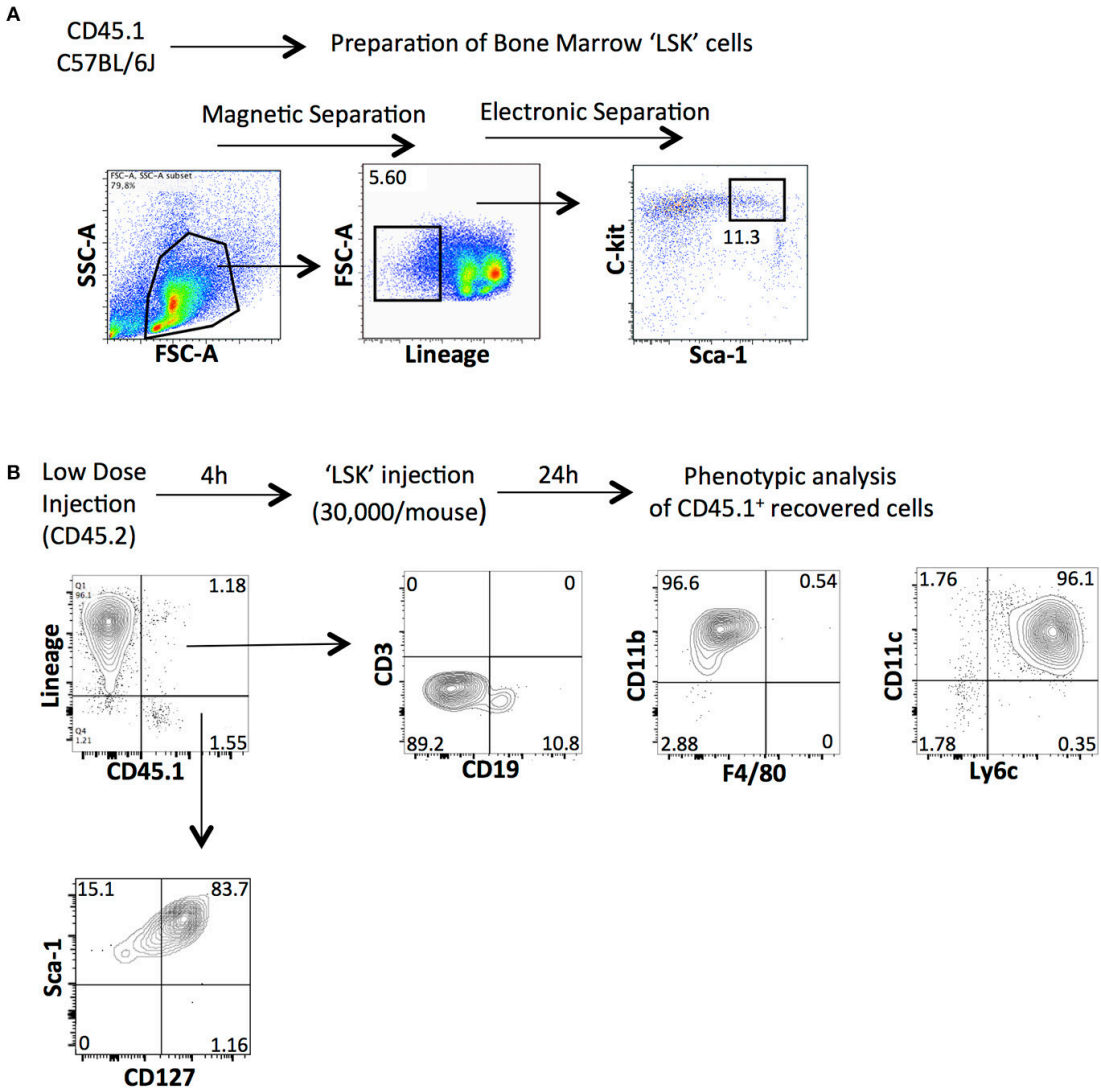
Intraperitoneal injection of BM-derived HSPCs contributes to the emergence of innate lymphoid-like cells in the peritoneal cavity of low-dose zymosan-injected mice. **(A)** Total bone marrow cells from congenic CD45.1 mice underwent magnetic and electronic cell separation in order to get pure hematopoietic stem cells (LSK). First, cells were selected based on the positive expression of CD117 (c-kit) and then stained for Lineage and Sca-1. A population of Lineage^−^c-kit^+^Sca-1^+^ was finally electronically sorted out. **(B)** CD45.2 C57BL/6J mice were injected intraperitoneally with a low-dose of zymosan and 4 h later the same recipients got 30,000 of LSK cells (i.p injection). Twenty-four hours post-injection, cells were recovered from the peritoneal cavity of injected mice and stained for several markers of mature immune cells (including CD3e, CD19, CD11b, F4/80, CD11c, and Ly6c) or markers for innate lymphoid-like cells such as Lineage, Sca-1 and CD127. (One representative experiment is shown from 3 independent ones).

To confirm our previous results suggesting that HSPCs derived from the bone marrow are needed to induce the emergence of ILCs in the peritoneal cavity of low-dose zymosan-injected mice, we performed adoptive transfer experiments. BM-HSPCs were magnetically selected based on the positive expression of c-kit (CD117) and then electronically sorted according to their expression of Lineage and Sca-1. The “LSK” cells (Lineage^−^ c-kit^+^ Sca-1^+^) cells were derived from congenic CD45.1 B6 mice and injected intraperitoneally into CD45.2 C57BL/6J mice 4 h post-injection of low-dose of zymosan (Figure 4A). Total cells were recovered from the peritoneal cavity of the recipient mice 24 h post-injection and were stained for markers of several types of immune cells including T cells (CD3e), B cells (CD19), dendritic cells (CD11c), macrophages (F4/80, CD11b) and markers of innate lymphoid cells (Lineage, CD127, and Sca-1). The congenic markers CD45.1 and CD45.2 were used to track exclusively our injected population. As expected, we show that the injected HSPCs as early as in 24 h they differentiate primarily in myeloid cells such as CD11b^+^ and CD11c^+^ subsets. The differentiation profile does not include any mature T cells or macrophages and only very few CD19^+^ B cells. Interestingly, we found that in the inflammatory environment induced by the low-dose injection of zymosan, HSPCs were capable of differentiating into a population of innate-like lymphoid cells since a population being negative for Lineage and positive for CD127 and Sca-1, could be recovered 24 h post-injection (Figure 4B).

### Pharmacological manipulation with an anti-inflammatory agent influences the migration and numbers of infiltrated ILCs at the site of inflammation

**Figure 5:**
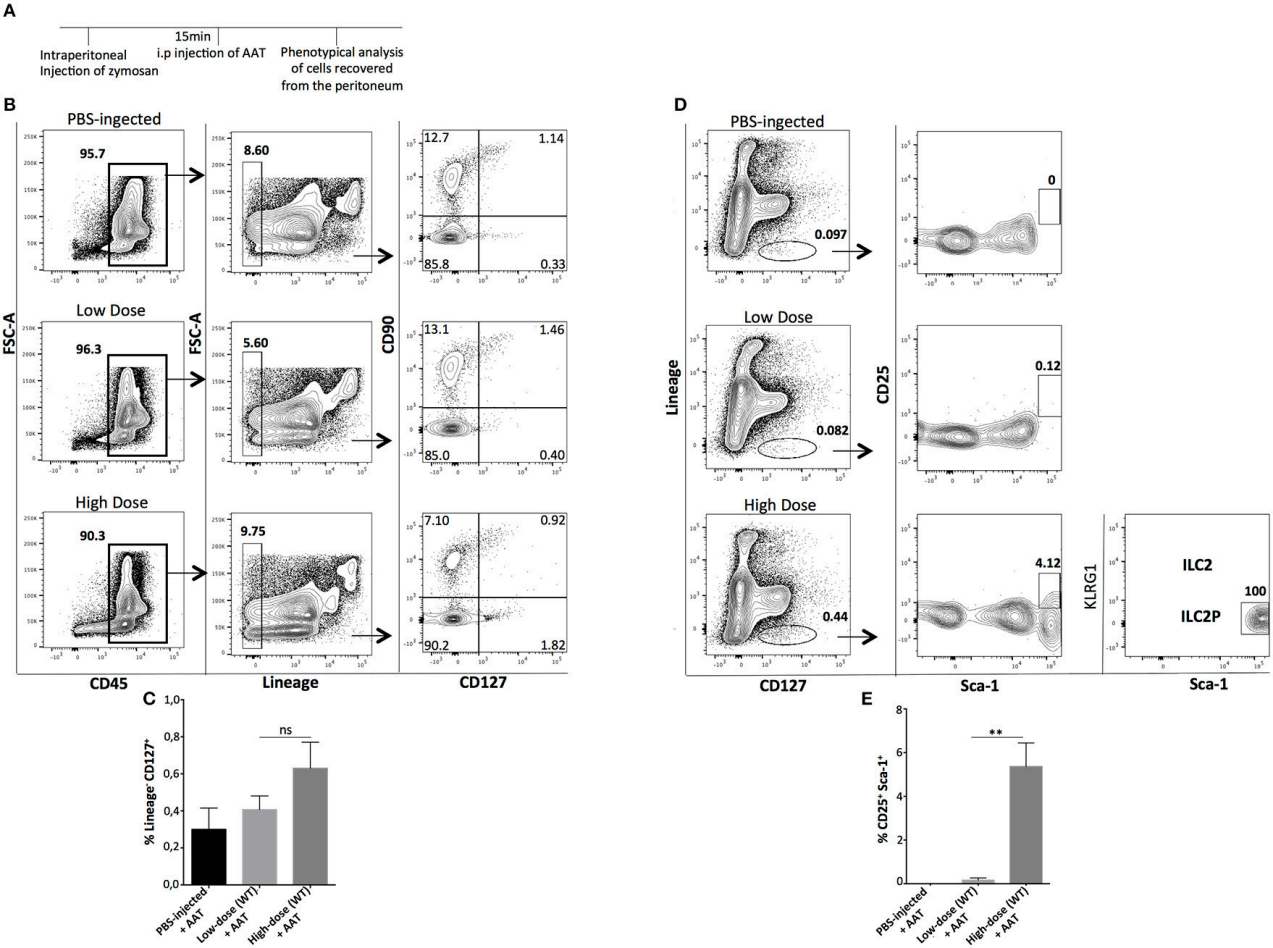
Treatment with Alpha-1-Antitrypsin inhibits the emergence of total ILCs in the peritoneal cavity of zymosan-injected mice. **(A)** C57BL/6J mice were injected intraperitoneally with low- or high-dose of zymosan and AAT treatment 15 min later. Peritoneal fluid from both zymosan-treated and PBS-injected (control) mice was purified and stained for markers of ILCs such as CD45, Lineage, CD127, and CD90. One representative flow cytometry analysis is shown from 4 independent experiments (*n* = 4). **(B)** Cells were stained for Lineage, CD127, CD25, Sca-1, and KLRG-1 in order to identify bone marrow-derived ILC2 and ILC2P cells (*n* = 4). **(C)** Statistical analysis of the frequency of Lineage^−^CD127^+^CD90^+^ cells (*n* = 4). **(D)** Flow cytometry gating strategy for ILC2 and their progenitor cells (ILC2P) in both mice injected with low- and high-dose of zymosan after treatment with AAT (*n* = 4). **(E)** Statistical analysis for the frequency of Sca-1^+^CD25^+^ cells within CD45^+^Lineage^−^ cells (*n* = 4). **p < 0.005.

Next, we sought to investigate whether a transient pharmacologic manipulation with alpha-1 anti-trypsin (AAT), an acute phase anti-inflammatory protein (25–27) can influence the presence, abundance, and phenotype of the recovered ILCs from the site of inflammation. Mice were injected i.p with low- or high-dose of zymosan and 15 min later they received an i.p injection with alpha-1 antitrypsin, a protein with well-known anti-inflammatory activity. The peritoneal fluid of the injected mice was analyzed 24 h post-injection (Figure 5A). Cells were stained for CD45, Lineage, CD127, and CD90, and found that AAT treatment reduced the appearance of ILCs at the site of inflammation (Figures 5B,C). Regarding the appearance of ILC2 in the peritoneum, we found that cells with a phenotype of ILC2P could still appear in the peritoneal cavity of high-dose zymosan-treated mice where, interestingly the emergence of the more mature BM-derived ILC2 has been lost (Figures 5D,E).

## Discussion

ILCs represent a recently discovered population of innate immune cells with some similarities to mature activated T lymphocytes. The development, function, and role of ILCs in health and disease have received considerable attention in both acute and chronic inflammatory conditions (28). Recent developments in the field suggest a strong role for ILCs in epithelial tissue homeostasis and clearance of viral infections, but the specific mechanism(s) involved are poorly understood (29, 30). In the present study, we sought to elucidate whether the strength of an inflammatory stimulus can determine the origins and differentiation of ILCs and subsets thereof as well as of HSPCs at the site of inflammation. However, identification of immature Lineage^−^ cells displaying characteristics of ILCs could be tricky due to a huge sensitivity in the gating strategy and should always be done with extreme caution.

Lineage reporter mice might be one option to detect and identify these immature cell populations. In our study, we have been very strict in identifying the Lineage^−^ cells, in order to avoid any mixture with more mature cells; nevertheless, there are still limitations of a definitive interpretation since it does not include target against Singlec, which stains for additional immune cell populations.

Both HSPCs and ILCs are locally present in the model of sterile peritonitis induced by i.p injection of the TLR2 agonist, zymosan. Injection of WT C57BL/6J mice with either low- or high-dose of zymosan induces the emergence of a population of mature ILCs (Lineage^−^CD45^+^CD127^+^CD90^+^) within the peritoneal cavity at 24 h post-injection.

We found that in WT zymosan-injected mice, the mature ILCs recovered from the peritoneal cavity, belong to the group 2 of ILCs since they express Gata-3 along with up-regulated levels of IL-5, a cytokine secreted mostly by this type of ILCs. In further support of the type 2 nature of recovered ILCs was the expression of ST2. ST2 belongs to the IL1R receptor family and IL-33 is its specific ligand. ST2^+^ mature ILC2 have been previously found in several tissues such as skin and lung, showing the importance of IL-33 response (31, 32). In our experiments, recovered ILCs have been positive for the expression of ST2, but not for IL17RB, suggesting differential requirements for IL-33 and IL-25 in this particular inflammatory environment.

To our knowledge, this is the first study, which focuses on the appearance of ILCs in the peritoneal cavity under inflammatory conditions, which raises the question whether the presence of ILCs is dependent upon migration of bone marrow-HSPCs to the site of inflammation.

Si et al. (20) demonstrated in the thioglycollate-induced sterile peritonitis model, that migration of HSPCs from the bone marrow to the site of inflammation is dependent upon chemokine receptor CCR2. In our experiments using CCR2 KO mice as recipients, the frequency of HSPCs in the peritoneal cavity of zymosan-injected mice is much lower compared to WT mice, validating these previously published results. However, the impact of CCR2 on the presence of ILCs has not been previously investigated.

Interestingly, in the absence of CCR2, a low-dose of zymosan was unable to induce the emergence of mature CD45^+^Lineage^−^CD127^+^ ILCs in the peritoneal cavity; that was not the case when a high-dose of zymosan was used. Taken together, these results suggest an involvement of HSPCs in the emergence of mature ILCs under certain conditions, since the presence of HSPCs was diminished in the peritoneal cavity of zymosan-injected CCR2 KO mice.

In our experimental setting, a high-dose treatment of zymosan could induce efficiently the emergence of mature ILCs regardless of the presence of BM-derived HSPCs, raising the possibility of alternative sources giving rise to mature ILCs at the site of inflammation. One such candidate that we further investigated in the present study could be bone marrow-derived ILC progenitors.

Except for the emergence of mature ILCs, we demonstrated that bone marrow-derived progenitor cells of type 2 could be also recovered from the peritoneal fluid of mice treated with either a high- or low-dose of zymosan, though in a dose-dependent manner. In low-dose treatment, the expression levels of cells displaying a phenotype of CD45^+^Lineage^−^CD127^+^CD25^+^Sca-1^+^ are much lower compared to the high-dose treatment. This might be explained by the fact that the need for specializing immune cells to emerge, participate, and fight against the inflammatory stimulus is different in the two conditions. The intensity of the inflammatory signals seems to affect differently both the emergence of HSCPs and progenitor cells of type 2 of ILCs. Interestingly, we see that bone marrow-derived ILC2 do not express CCR2 so we suggest that CCR2 might be involved indirectly in the emergence of progenitor cells of ILCs at the site of inflammation.

In CCR2 KO mice, a low-dose zymosan injection was unable to induce the emergence of progenitor cells suggesting that HSPCs might play a role either in the trajectory of ILC2P from the bone marrow to the site of inflammation, or *in situ* differentiation into ILC2P. In contrast, when CCR2 KO mice are treated with a high-dose of zymosan, regardless the presence of HSPCs, both mature ILCs and precursors of ILC2 were recovered. Thus, the emergence of ILCs into the high-dose of zymosan, induced by a hyper-inflammatory stimulus, is an HSPC-independent process. In order to support further our evidence that the ILCs emerged in the peritoneal cavity of low-dose zymosan are dependent on BM-derived HSPCs, we performed an adoptive transfer of bone marrow-derived HSPCs and studied their differentiation potential in response to zymosan. Our results indicate that this inflammatory environment allowed HSPCs to differentiate primarily into myeloid cells. Interestingly, we found that they were also able to give rise to innate-like lymphoid cells since a population of Lineage^−^CD127^+^Sca-1^+^ cells could also be recovered. Ideally, to avoid competition between host and injected HSPCs, CCR2 KO mice as recipient mice should have been used. Additionally, a BM-chimera might be useful as well to study into more depth HSPCs dependence of ILCs.

To further elucidate the importance of the inflammatory environment *per se* for the attraction of mature ILCs, an anti-inflammatory drug was used. Treatment of mice with alpha-1 anti-trypsin (AAT) 15 min after injection of either low- or high-dose of zymosan, led to the blockade of the migration of HSPCs into the peritoneal cavity resulting in a loss of even mature ILCs in mice injected with the low-dose of zymosan. Contrary, in mice injected with high-dose, progenitor cells of type 2 of ILCs could be still recovered, but not mature ILC2 cells, suggesting that the single injection of AAT is insufficient to stop the strong inflammatory responses induced by hyper-inflammatory stimuli.

Taken together, the above experiments suggest that manipulation of early hematopoietic cells might open new perspectives on how we can prevent or stop inflammatory responses.

## Materials and methods

### Mice

Mice are housed in individually ventilated cages at the Association for Assessment and Accreditation of Laboratory Animal Care (AAALAC)-accredited, specific-pathogen-free BIDMC CLS animal facility. Mouse use and care are according to the guidelines established by the Animal Care Committee at Beth Israel Deaconess Medical Center. C57BL/6J (CD45.2 and/or CD45.2) wild-type and CCR2 Knock-out mice were purchased from the Jackson Laboratory (Bar Harbor, ME). Female mice between 8 and 12 weeks of age used for the studies.

### Isolation of bone marrow cells

Bone Marrow cells were flushed from the tibia and femur with HyClone RPM1-1640 Medium supplemented with 2.05 mM L-Glutamine and 10% FCS. Cells were filtered through a nylon screen (70 mm) to obtain a single-cell suspension. Red blood cells were depleted with ACK lysis buffer (150 mM NH_4_Cl, 1 mM KHCO_3_, 0.1 mM EDTA).

### Extraction of RNA and qRT-PCR

Extraction of RNA from sorted cells was performed with use of Qiagen Rneasy Mini Kit following manufacturer instructions. Quantitative real-time PCR (qRT-PCR) was performed to detect mRNA expression for gata-3 and il-5 (TaqMan). The primers used for qRT-PCR are shown in Table 1.

**Table 1:** Primers used for qPCR experiments.

**Protein**	**TaqMan Assay ID**
IL-5	Mmoo439646_m1
Gata-3	Mmoo484683_m1

### Zymosan-induced peritonitis

Peritonitis was induced by an i.p injection of zymosan (Zymosan A, Sigma) into wild-type (WT) and CCR2 KO mice as described. Peritoneal cells were collected 24 h later by flushing the peritoneal cavity with 3 ml of PBS 1X for three times. After red blood cell lysis, peritoneal cells were washed with staining buffer (PBS 2% FCS), and 3 × 10^6^ cells were incubated with anti-mouse CD16/CD32 for 30 min to block Fc receptors. After washing, the peritoneal cells were incubated with antibodies for several lineage markers, as described above, for 30 min. Cells were washed twice with staining buffer and analyzed by flow cytometry, as described above.

### Alpha-1-antitrypsin

Aralast (α-proteinase inhibitor, human; Shire Plc), an AAT product obtained from human blood, is a concentrate of this serum serine-protease inhibitor. Mice were injected i.p with 60 mg/kg of AAT 15 min after the zymosan injection.

### Facs analysis and cell separation/sorting

The following monoclonal antibodies were used: Anti-Lineage, a cocktail containing a concentration optimized mix of the following antibodies: CD3e, Ly6G/Ly6c, CD11b, CD45R/B220, TER-119 (BioLegend, San Diego, CA). Monoclonal Antibodies against CD117 (c-kit), Sca-1, CD127 (IL7R), CD45, KLRG-1, CD25, CD90, CD34, ST2, IL17RB, CCR2, T-bet, Gata-3, and RORgt were purchased from BioLegend. Live/Dead Yellow Fixable Stain, Fixable Viability Dye eFluor 780 or DAPI were utilized to assess viability. Data acquisition and/or cell sorting was performed on a special-order 5-laser BD LSRII flow cytometer, Beckman Coulter Gallios, or BD FACS Aria or Aria II instrument. Purity after sorting was routinely >95%. The analysis was performed using FlowJo software. Mean fluorescence intensities (MFIs) were calculated using the geometric mean of the appropriate fluorescence channel in FlowJo. Expansion Indices were determined using the embedded FlowJo algorithm. For the magnetic separation of hematopoietic stem cells, we used the EasySep mouse CD117 (c-kit) positive selection kit from StemCell Technologies (18757) and we followed the protocol provided by the company.

### Intracellular staining process

Intracellular staining for the expression of transcription factors was performed with the use of a kit from eBioscience (Foxp3/Transcription factor staining buffer set). Cells underwent fixation and permeabilization following the protocol provided by the company.

### Statistics

Statistical analyses were performed with Prism software (GraphPad, La Jolla, CA). Means of two groups were compared by an unpaired *t*-test. Means of three or more groups were compared using a one-way ANOVA with Bonferroni's post-test. All error bars are ±S.D Values of *p* < 0.05 were deemed statistically significant.

## Ethics statement

This study was carried out in accordance with the principles and recommendations of the Institutional Animal Care and Use Committee (IACUC) of Beth Israel Deaconess Medical Center. This institution's Institutional Animal Care and Use Committee reviewed and approved the protocol on May 5, 2017 (RN-150D).

## Author contributions

SK designed and performed the experiments, interpreted the data, and wrote the manuscript. TBT, PK, ET, LM, LSL and EK performed experiments and analyzed data. TBS and MK designed the project. EK, TBS and MK provided critical review of the data and manuscript.

### Conflict of interest statement

The authors declare that the research was conducted in the absence of any commercial or financial relationships that could be construed as a potential conflict of interest. The reviewer, RKR declared a shared affiliation, with no collaboration, with the authors to the handling Editor.
