# Supplementary figures and images for: Hematopoietic Stem/Progenitor Cell Dependent Participation of Innate Lymphoid Cells in Low-Intensity Sterile Inflammation
Source: Front Immunol. 2018 Sep 5;9:2007. doi: 10.3389/fimmu.2018.02007 (PMC6134892; doi:10.3389/fimmu.2018.02007)

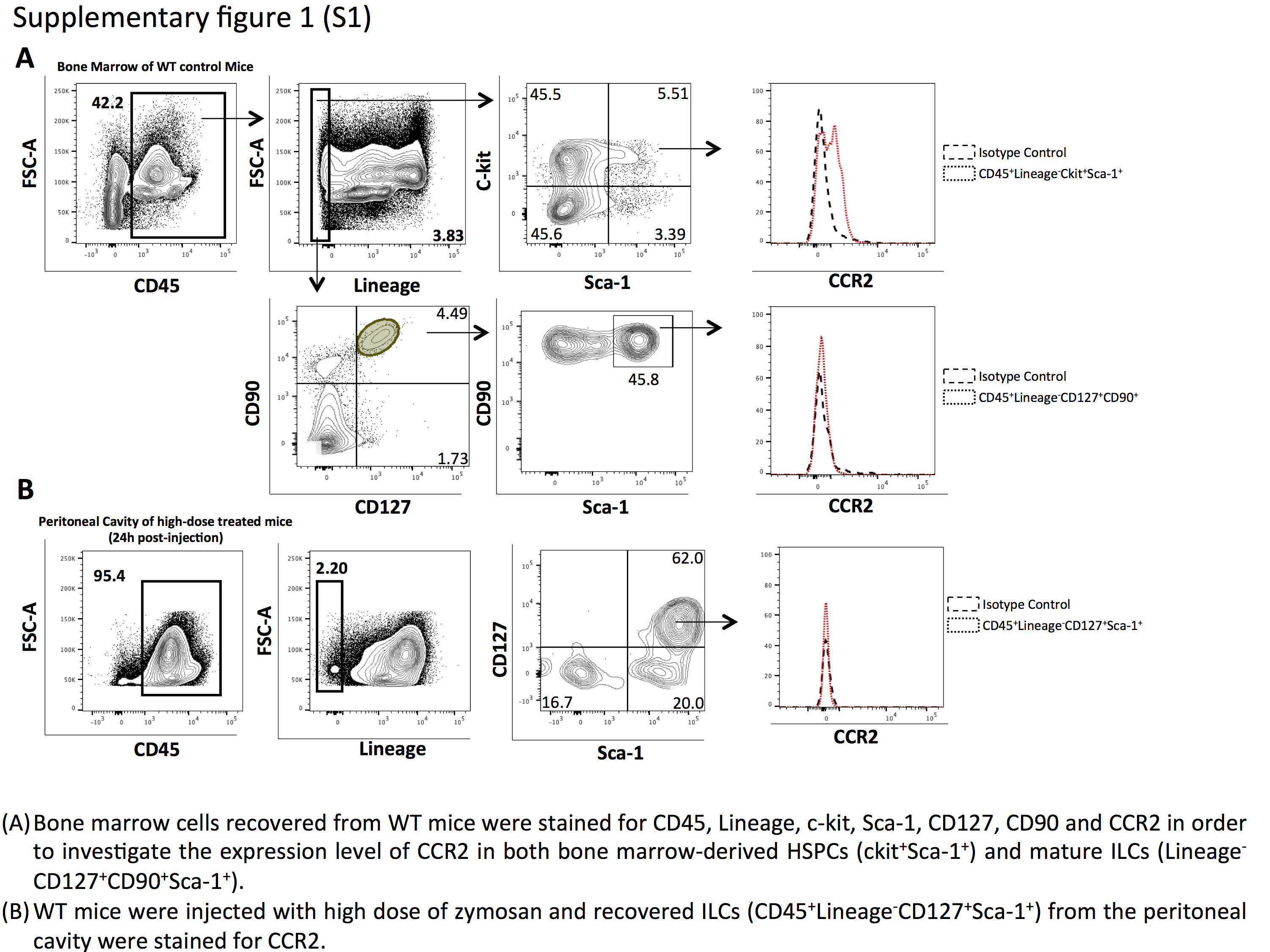

Supplement: Supplementary file 1 [file Image_1.tiff]

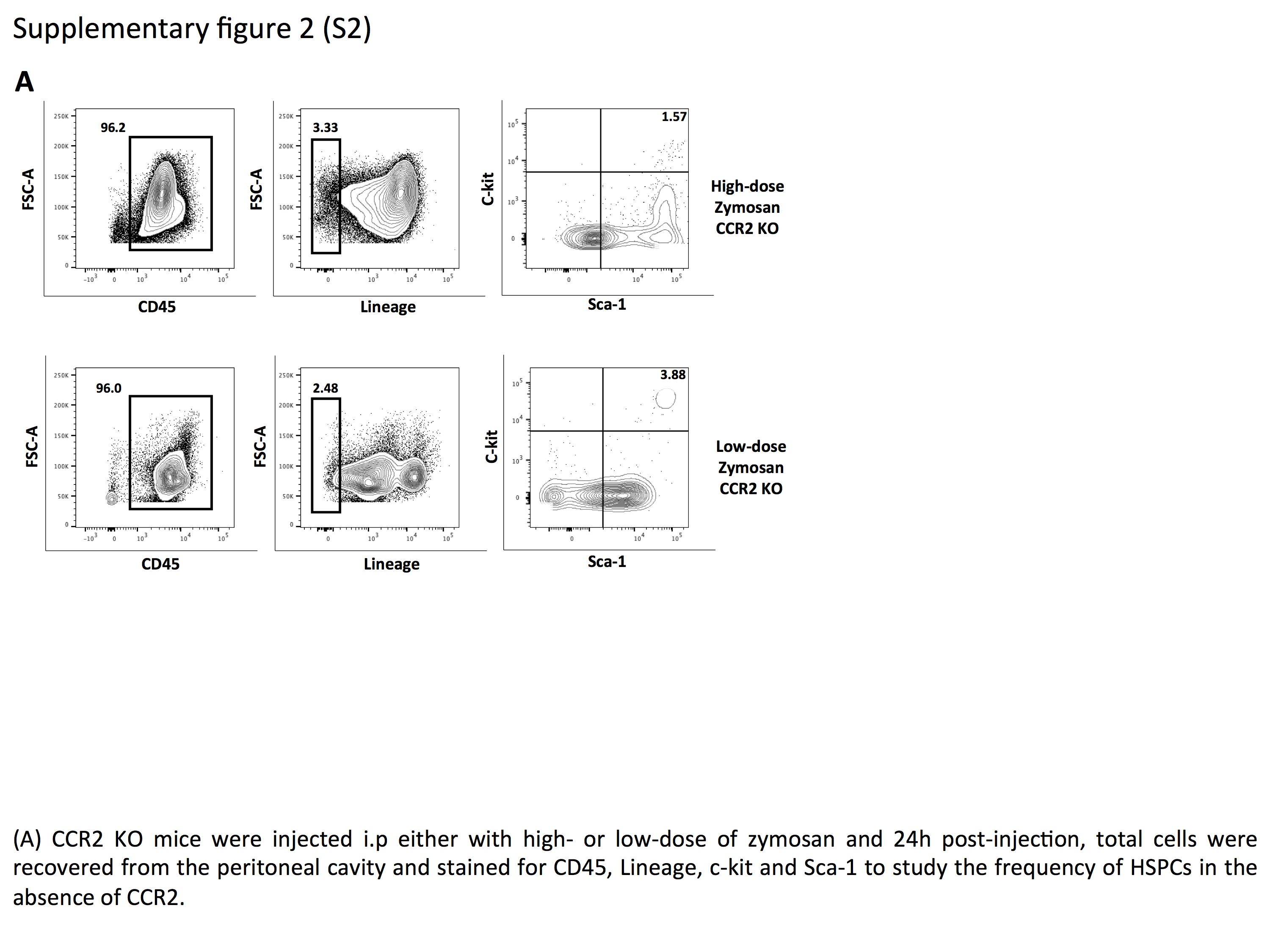

Supplement: Supplementary file 2 [file Image_2.tiff]

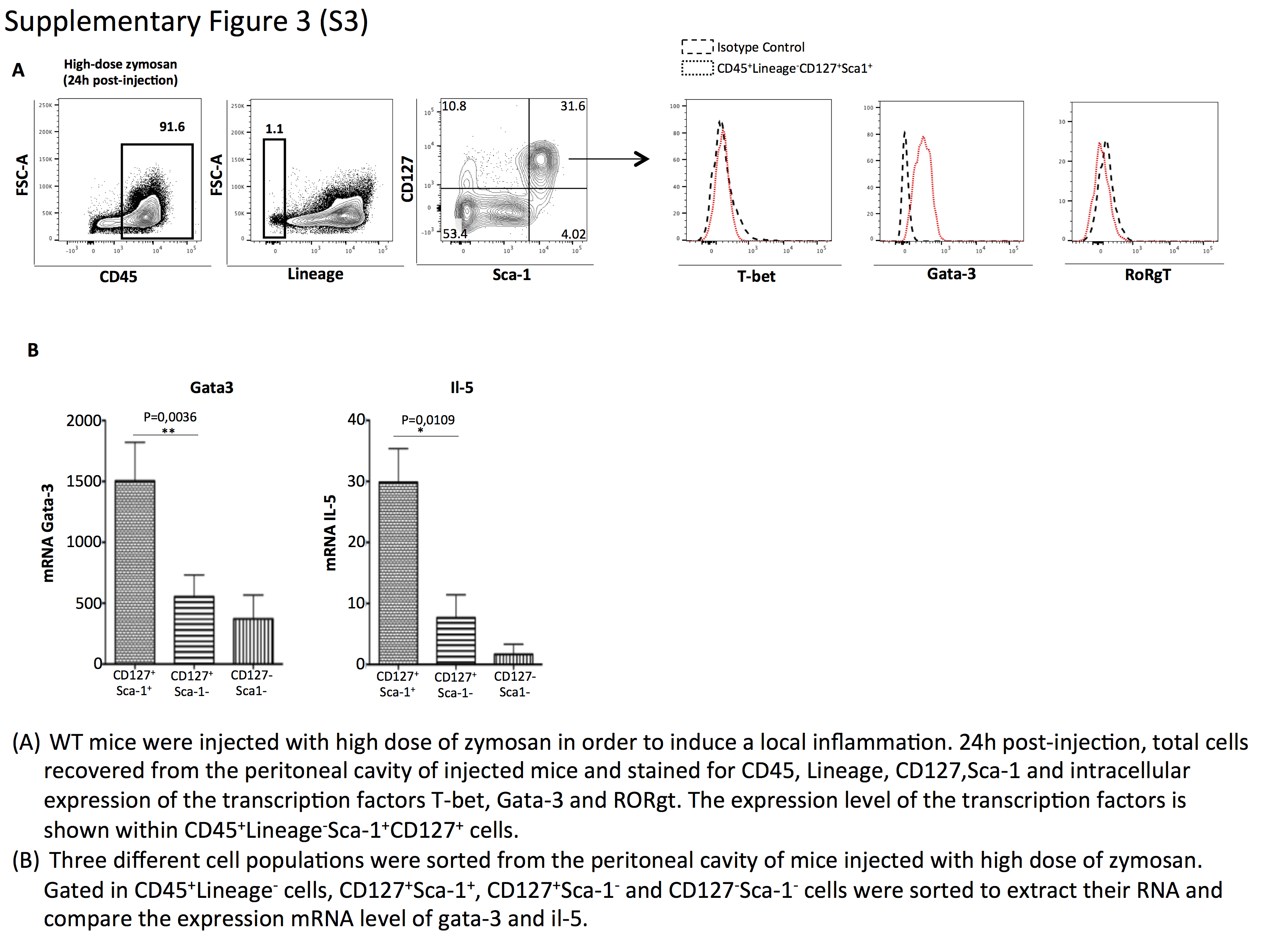

Supplement: Supplementary file 3 [file Image_3.tiff]

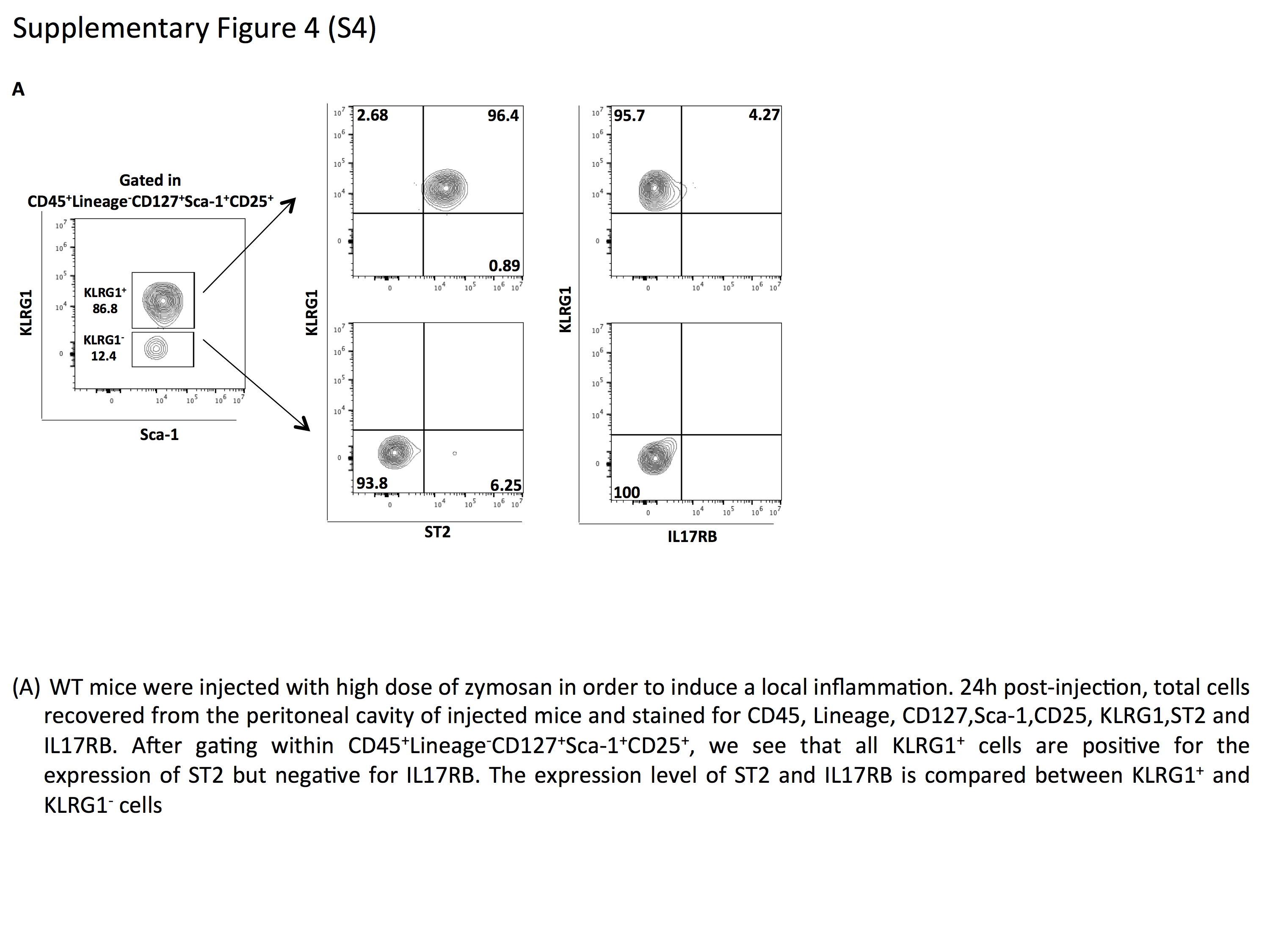

Supplement: Supplementary file 4 [file Image_4.tiff]
